# Correlation of progesterone levels on the day of oocyte retrieval with basal hormonal status and the outcome of ART

**DOI:** 10.1038/s41598-020-79347-2

**Published:** 2020-12-18

**Authors:** L. Tulic, I. Tulic, J. Bila, Lj Nikolic, J. Dotlic, M Lazarevic-Suntov, I. Kalezic

**Affiliations:** 1 Department of In Vitro Fertilization, Clinic for Gynecology and Obstetrics, Clinical Center of Serbia, Faculty of Medicine, University of Belgrade, Dr Koste Todorovica 26, 11000 Belgrade, Serbia; 2 Faculty of Medicine, University of Belgrade, Dr Subotica 8, 11000 Belgrade, Serbia; 3 Clinic for Gynecology and Obstetrics, Clinical Center of Serbia, Dr Koste Todorovica 26, 11000 Belgrade, Serbia; 4 Department of Biochemistry, Clinic for Gynecology and Obstetrics, Clinical Center of Serbia, Dr Koste Todorovica 26, 11000 Belgrade, Serbia

**Keywords:** Endocrinology, Medical research

## Abstract

This study aimed to assess whether basal hormonal levels can predict the levels of progesterone (P4) on the day of oocyte retrieval (OR) and examine the impact of P4 levels on the day of OR on the outcome of assisted reproduction. One hundred sixty-four patients that were enrolled in the assisted reproduction procedure were classified according to their P4 levels on the OR day (< 2 ng/ml vs. ≥ 2 ng/ml). Patients who had P4 levels < 2 ng/ml had significantly higher follicle stimulating hormone (FSH) levels and significantly lower anti-Mullerian hormone (AMH) levels. More than half of patients with P4 levels < 2 ng/ml on the OR day got pregnant and delivered healthy infants. There was a significant correlation between lower FSH values and higher P4 values at OR and between higher AMH values and higher P4 values on the day of OR. Regression analysis showed that high FSH levels are the most important factor that can reliably imply lower P4 levels on OR day. Our study confirmed that lower basal FSH levels can predict the levels of P4 on the OR day. Moreover, lower levels of P4 on the day of OR are associated with a positive outcome in assisted reproduction.

## Introduction

Multiple studies have indicated that some patients have progesterone (P4) elevation on the day of human chorionic gonadotropin (hCG) administration during controlled ovarian stimulation (COS) of assisted reproductive technology (ART)^1–3^. Although the exact mechanism is unclear, this elevation has been shown to negatively impact the outcome of the ART procedure. Researchers believe that preovulatory P4 elevation can be caused by several mechanisms: accumulation of P4 production from multiple developing follicles during COS^3^, excessive amount of exogenous gonadotropins (GT)^4^, premature luteinization^5^, poor ovarian response with increased luteinizing hormone (LH) sensitivity^3^, total dose of administered GTs^4,6^, and duration of ovarian stimulation^7^.

Some studies have shown that premature P4 elevation is a cause of reduced pregnancy rates as it affects endometrial receptivity (i.e., endometrial maturation), leading to asynchrony between the endometrium and the embryo^8^, while other investigations have found that oocyte quality may be compromised by premature P4 elevation^9,10^. Despite effective suppression with gonadotropin-releasing hormone (GnRH) analogs, P4 elevation is reported in 5–53% of stimulated cycles^3–7^.

Several studies have examined the effect of P4 elevation on the day of hCG trigger in cycles with agonists and antagonists, but only a few studies have examined the effect of P4 levels on the day of oocyte retrieval (OR) on the outcome of ART^1–3,11,12^.

Previous studies on ART procedures have shown different relationships between steroid hormones and the outcome of the ART procedures. Basal hormonal status is generally determined before the commencement of ovarian stimulation. Levels of anti-Mullerian hormone (AMH), basal follicle stimulating hormone (FSH), and estradiol (E2) are widely used as a prognostic test for ovarian reserve, while basal P4 levels is used to predict the outcome of the ART procedure^3–7^.

The study aimed to determine P4 levels on the day of OR, 34–36 h post hCG administration, and investigate whether P4 levels at this time affect the outcome of the ART procedure and subsequently the pregnancy rate. Moreover, since FSH, E2, P4, and AMH are considered predictors of ovarian response and ART outcome and because little is known of the variables that could identify patients at risk of P4 elevation, this study also aimed to determine whether basal hormonal levels can predict the levels of P4 on the day of OR.

## Results

### Patients’ characteristics and basal hormonal levels according to P4 levels

The study evaluated 164 patients undergoing an ART procedure (OR and embryo transfer) in our clinic. Out of them 44.5% were in the P4-low group (< 2 ng/ml) and 55.5% in the P4-high group (≥ 2 ng/ml). The baseline characteristics of the patients are shown in Table 1. Patients on average were 34.66 ± 3.39 years old, the mean BMI was 22.23 ± 2.65, and their infertility generally lasted for 4.86 ± 2.61 years (range 1 to 17 years). The mean P4 values on OR day were 1.26 ± 0.46 ng/ml in the P4-low group, while in the P4-high group the average P4 was 3.74 ± 1.62 ng/ml. No significant differences were found in LH, P4, and E2 levels or in GT dose regarding the P4 values on the day of OR, while patients from the P4-low group had significantly higher FSH levels and significantly lower AMH levels (Table 1).

**Table 1:** Patients’ characteristics and basal hormonal levels regarding P4 levels on the day of OR.

Tested parameters		Mean ± SD	Median	95% CI	Min	Max	p
				Lower–Upper			
Female age (years)	P4-low	35.36 ± 3.09	35.00	34.63–36.08	26.00	40.00	0.065
	P4-high	34.11 ± 4.04	34.50	33.27–34.95	21.00	41.00	
	Total	34.66 ± 3.69	35.00	34.10–35.23	21.00	41.00	
BMI (kg/m^2^)	P4-low	22.39 ± 2.50	21.75	21.81–22.98	18.00	29.40	0.486
	P4-high	22.10 ± 2.77	21.35	21.53–22.68	18.20	29.40	
	Total	22.23 ± 2.65	21.65	21.82–22.64	18.00	29.40	
Infertility duration (years)	P4-low	4.69 ± 2.54	4.69	4.10–5.28	1.00	13.00	0.438
	P4-high	4.99 ± 2.67	4.99	4.43–5.55	2.00	17.00	
	Total	4.86 ± 2.61	4.00	4.45–5.26	1.00	17.00	
Basal P4 (ng/ml)	P4-low	1.71 ± 1.86	1.00	1.23–2.18	0.40	10.6	0.644
	P4-high	1.57 ± 1.74	1.67	0.96–1.94	0.18	12.5	
	Total	1.51 ± 0.80	1.50	1.39–1.63	0.27	4.30	
Basal FSH (mIU/ml)	P4-low	7.71 ± 2.46	7.38	7.13–8.30	3.00	14.80	0.015*
	P4-high	6.88 ± 2.43	6.45	6.37–7.39	2.60	15.00	
	Total	7.24 ± 2.47	6.85	6.86–7.63	2.60	15.00	
Basal LH (mIU/ml)	P4-low	5.18 ± 1.95	4.75	4.70–5.66	0.40	9.30	0.252
	P4-high	5.13 ± 3.09	4.65	4.48–5.77	0.40	25.20	
	Total	5.15 ± 2.66	4.70	4.73–5.57	0.40	25.20	
Basal E2 (pg/ml)	P4-low	44.90 ± 20.95	40.00	39.90–49.89	14.50	95.00	0.755
	P4-high	42.88 ± 18.23	40.00	38.99–46.77	10.00	100.00	
	Total	43.78 ± 19.45	40.00	40.71–46.85	10.00	100.00	
AMH (ng/l)	P4-low	2.20 ± 2.53	1.42	1.57–2.82	0.10	12.00	0.038*
	P4-high	2.82 ± 2.62	1.89	2.26–3.39	0.10	14.30	
	Total	2.55 ± 2.59	1.80	2.13–2.97	0.10	14.30	
GT dose	P4-low	2222.57 ± 546.87	2250.00	2094.1–2351.1	1275.00	3825.00	0.800
	P4-high	2190.52 ± 535.01	2175.00	2076.5–2304.5	900.00	4125.00	
	Total	2205.03 ± 538.93	2175.00	2120.6–2289.5	900.00	4125.00	

*BMI* body mass index, *FSH* follicle stimulating hormone, *LH* luteinizing hormone, *E2* estradiol, *P4* progesterone, *AMH* anti-Mullerian hormone, *GT* gonadotropins, *ART* assisted reproduction technologies.

*p < 0.05

### Clinical features and procedure outcome depending on P4 level

In the overall sample, the most common causes of infertility were unknown factors (30.5%) and the male factor (28%). In the P4-high group of patients, the most common cause of infertility was the male factor, while the ovarian factor was the most frequent cause in patients from the P4-low group. Nevertheless, there was no significant difference in the cause of infertility regarding P4 levels on the day of OR (Table 2). The average number of all retrieved oocytes, as well as the number of mature oocytes and fertilized oocytes, was significantly higher in the P4-high group. In contrast, the average rate of fertilization was significantly lower in the P4-high group. Although the embryos were mostly of grade A quality in the P4-high group on the OR day, the difference in embryo quality was not significant between P4 level groups. Moreover, there were no significant differences in the number of transferred embryos regarding the two groups of patients (Table 2).

**Table 2:** Clinical features and procedure outcome depending of P4 level on the day of OR.

Investigated parameters		Whole sample		P4-low (< 2 ng/ml)		P4-high (≥ 2 ng/ml)		Between groups p
		Number	%	Number	%	Number	%	
Cause of infertility	Male factor	46	28.0	18	39.1	28	60.9	0.871
	Tubal factor	31	18.9	13	41.9	18	58.1	
	Ovarian factor	30	18.3	15	50.0	15	50.0	
	Unexplained	50	30.5	24	48.0	26	52.0	
	Combined factor	7	4.3	3	42.9	4	57.1	
Stimulation protocol	Long agonist	18	11.0	4	22.2	14	77.8	0.044*
	Short antagonist	146	89.0	69	47.3	77	52.7	
Insemination method	IVF	69	42.1	58	52.7	59	57.8	0.040*
	ICSI	48	29.2	20	18.2	28	27.5	
	Combined	47	28.7	32	29.1	15	14.7	

Number of oocytes								
	Mean	SD	Mean	SD	Mean	SD	p	
Oocytes no	8.97	6.47	7.07	6.42	10.49	6.14	≤ 0.001*	
Mature oocytes no	7.50	5.40	5.95	5.57	8.75	4.95	≤ 0.001*	
Fertilized oocytes no	5.08	4.41	4.44	4.70	5.59	4.12	0.017*	
Fertilization rate	71.36	27.66	77.23	24.36	66.65	29.34	0.021*	
Transferred oocytes	1.96	0.15	2.09	0.11	1.84	0.73	0.106*	

Embryo quality								
		Number	%	Number	%	Number	%	p
Class A embryos		93	56.7	37	39.8	56	60.2	0.163
Class B embryos		100	61.0	46	46.0	54	54.0	0.632
Class AB embryos		28	17.1	14	50.0	14	50.0	0.521
Class C embryos		33	20.1	16	48.5	17	51.5	0.607
**Outcome measures**								
Outcome of ART	Non pregnant	76	46.3	25	32.9	51	67.1	0.005*
	Pregnant	88	53.7	48	54.5	40	45.5	
Outcome of pregnancy	Biochemical	12	13.6	4	8.3	8	20	0.016*
	Miscarriage	5	5.7	3	6.3	2	5	
	Delivery	71	80.7	41	85.4	30	75	

*p < 0.05

Patients on the short stimulation protocol had significantly lower levels of P4 on the OR day than those on a long stimulation protocol. It was noticed that patients from the P4-high group underwent more often a long stimulation agonist protocol (Table 2). The odds for procedure success of subjects from the P4-low group stimulated with a short protocol was almost three times higher than those stimulated with a long protocol (OR = 2.17; 95% CI 1.06–4.42). Patients undergoing IVF or ICSI techniques had lower levels of P4 on the OR day compared to those who underwent the combined technique of insermination (Table 2). We found a statistically significant difference in the levels of P4 on the day of OR in relation to the positive outcome of the ART procedure as well as regarding the outcome of pregnancy. More than half of the patients from the P4-low group on the OR day got pregnant after ART procedure. In addition, 80.7% of all investigated women successfully delivered an infant (Table 2).

### Correlation of P4 on the OR day with patient and procedure characteristics, basal hormonal status, and regression analysis

By examining the association between P4 and basal hormone levels, a statistically significant correlation was found between lower FSH values and higher P4 values on OR day both directly (i.e., continuously) and with P4 ≥ 2 ng/ml on the OR day (Table 3). Furthermore, higher AMH values were significantly correlated with higher P4 values on the day of OR. Higher P4 values on OR day were also correlated with long stimulation protocol and the use of the IVF/ICSI combined technique (Table 3).

**Table 3:** Correlation of P4 on the OR day with patient and procedure characteristic and basal hormonal status.

Investigated parameters		P4 continuous	P4 groups low/high (< 2 and ≥ 2 ng/ml)
Female age	r/Ro	− 0.124	− 0.231
	p	0.121	0.056
Age groups (< 29, 30–35, ≥ 36 years)	Ro	− 0.117	− 0.140
	p	0.135	0.073
BMI continuous	r/Ro	− 0.124	− 0.094
	p	0.115	0.231
BMI groups (< 25 and ≥ 25)	Ro	0.010	− 0.011
	p	0.900	0.889
Smoking status	Ro	0.055	− 0.010
	p	0.485	0.904
Infertility duration (years)	R	0.109	0.065
	P	0.166	0.407
Infertility cause	R	− 0.034	− 0.086
	P	0.664	0.275
Stimulation protocol	R	0.177	0.157
	P	0.024*	0.044*
Insemination method	R	0.174	0.196
	P	0.026*	0.012*
**Basal hormonal levels**			
FSH	r/Ro	− 0.230	− 0.194
	p	0.003*	0.014*
LH	r/Ro	− 0.035	− 0.092
	p	0.664	0.254
E2	r/Ro	− 0.015	− 0.025
	p	0.852	0.756
P4	r/Ro	− 0.011	− 0.024
	p	0.891	0.776
AMH	r/Ro	0.271	0.170
	p	0.001*	0.037*
GT dose	r/Ro	− 0.019	− 0.020
	p	0.808	0.801

*BMI* body mass index, *FSH* follicle stimulating hormone, *LH* luteinizing hormone, *E2* estradiol, *P4* progesterone, *AMH* anti-Mullerian hormone, *GT* gonadotropins, *ART* assisted reproduction techniques.

*p < 0.05

Univariant regression analysis suggested that basal FSH level could significantly predict P4 levels, as well as combined ART technique and stimulation protocol. Furthermore, preformed multivariate regression analysis showed that high FSH levels on OR day are the most important factor for reliably imply on lower P4 levels. The regression analysis is presented in Table 4.

**Table 4:** Regression analysis of potential P4 level on the OR day predictors.

Predictors	Univariant				Multivariant			
	OR	p	95% C.I		OR	p	95% C.I	
			Lower	Upper			Lower	Upper
Female age	0.62	0.059	0.38	1.00				
BMI continuous	0.96	0.484	0.85	1.08				
Smoking status	0.95	0.903	0.44	2.05				
FSH	0.87	**0.037**	0.76	0.99	0.87	0.050*	0.77	1.00
P4	0.95	0.643	0.79	1.15				
LH	0.99	0.903	0.88	1.12				
E2	0.99	0.518	0.98	1.01				
AMH	1.11	0.146	0.97	1.27				
GT dose	1.00	0.708	1.00	1.00				
Infertility duration	1.29	0.437	0.68	2.43				
Infertility cause	0.89	0.269	0.73	1.09				
Stimulation protocol	3.14	0.050*	0.99	9.98	2.85	0.079	0.88	9.18
Insemination method	2.09	0.042*	1.10	4.27	1.45	0.061	1.00	3.37

*BMI* body mass index, *FSH* follicle stimulating hormone, *LH* luteinizing hormone, *E2* estradiol, *P4* progesterone, *AMH* anti-Mullerian hormone, *GT* gonadotropins, *ART* assisted reproductive technologies.

*p < 0.05

## Discussion

The impact of preovulatory P4 rise on the outcome of ART procedures remains controversial. While some studies have not shown a negative effect on ART outcome^11,12^, other studies associated unsuccessful ART with elevation of P4 levels^1–3^. Possible explanations for the discrepancies in the findings are the use of retrospective study design, the use of different protocols of controlled ovarian stimulation, and different cutoff levels for P4at the time of data analysis. Some studies imprecisely define references for elevated serum P4 levels, and there is variation in the statistical methods used to estimate specific circulating P4 limit values and in the precision of P4measurements that use different immunoassays^12,13^. The cutoff of P4 absolute concentrations on the day of hCG administration generally ranges from 0.8 to 2 ng/ml^14–16^. However, in recently published studies, it has often been adjusted to 1.5 ng/mL^17^. Based on the ROC analysis we performed, 2 ng/mL was confirmed as the P4 cutoff level for achieving pregnancy. Therefore, this P4 level was used for all further analyses.

Cycles using GnRH agonists (GnRHa) have been the most studied, while the effect of P4 levels on the day of hCG trigger or at other critical points during stimulation with GnRH antagonists (GnRHant) has been less examined. An increase in P4 levels was observed in GnRHa protocols despite down-regulation of the pituitary gland, while slightly lower P4 levels in patients treated with GnRHant were explained by lower steroidogenic activity of granulose cells or half-life of the antagonist (about 20 h), which leads to rapid recovery of pituitary suppression^5,18^.

In this study, a short protocol with GnRH antagonists and a long protocol with GnRH agonists were used, and although the number of patients with a long stimulation protocol was smaller, we found that an elevation in P4 levels on the day of OR was more frequent in the long stimulation protocol. Our findings can also be explained to some extent by the fact that the long protocol was more commonly used in younger patients where we received more oocytes. Moreover, although we found that higher P4 levels on the day of OR were more prevalent in the younger patients, these findings were not significant. This is also consistent with the findings of a meta-regression analysis by Venetis et al.^19^, which showed that the protocol with GnRH antagonists was associated with lower rates of P4 rise compared to protocols with GnRH agonists, independent of the P4 threshold. However, a study by Huang et al.^2^, which examined elevated P4 in both the long and short protocols, showed that P4 levels were higher in the short protocol than in the long stimulation protocol. They explained these differences in the findings based on the fact that the characteristics of patients in the short and long protocol differed; more poor responders were on the short stimulation protocol, the cycle characteristics of the two protocols differed, and the number of patients on the short protocol was higher, which could have been confusing^2^.

Some studies have not found an adverse effect of P4 levels on the outcome of ART procedures and on pregnancy rates^12,15^; however, there are several publications that have found a significant negative association between P4 levels on the hCG trigger day and the success of ART procedures^4,20,21^. In a study of 2566 patients undergoing the ART procedure, Huang et al.^2^ found a negative correlation between elevated P4 and live birth rate (the cutoff value was 1.2 ng/ml in long protocols and 2.0 ng/ml in short protocols). Another meta-analysis by Venetis et al.^19^ showed that P4 elevation on the day of hCG administration was associated with significantly reduced pregnancy rates after a fresh embryo transfer. A recent publication showed that the live birth rates were significantly lower in patients with both low and high P4levels on the day of hCG administration^22^.

All of these studies measured P4 levels on the day of hCG administration, but a study by Niu et al.^23^ examined P4 levels on the day of OR in cycles with GnRH agonists (long and short protocol) and found that P4 levels correlated with the number of oocytes and embryos, but can’t predict the outcome of the pregnancy. In a prospective study of 186 women, Nayak et al.^24^ examined the prediction of ART success depending on P4 level on the day of OR in patients on a short stimulation protocol with GnRHant. There was no correlation of basal P4 andP4 levels on the day of antagonist initiation with P4 on the day of hCG administration. Patients with a P4 rise on the day of OR had lower pregnancy and implantation rates in their study^24^. In this study, a statistically significant difference in the level of P4 on the day of OR compared to pregnancy rates was found. Patients with P4 levels < 2 ng/ml had higher pregnancy rates. In terms of pregnancy outcome, there were fewer miscarriages in this group and the delivery rate was higher. This study demonstrates the predictive effect of lower P4 values on OR day and the positive outcome of the ART procedure.

In terms of basal hormones, we found lower AMH levels in women with lower P4 (P4 < 2 ng/ml) on OR day as well as a direct correlation between these parameters. However, AMH has not been shown to predict P4 rise on the day of oocyte aspiration. Our finding is consistent with a recent study by Kavoussi et al.^25^, who also did not find a predictive value of AMH in P4 rise. Studies have shown that AMH can predict the response to ovarian stimulation as well as the number of achieved oocytes and embryos; however, its correlation with P4 rise has not been extensively studied^25^.

Studies have shown that the level of basal P4 can influence the outcome of the ART procedure^26,27^. The association of basal P4 with P4 elevation on the day of hCG was also examined previously^28^. Although this study did not confirm the adequate predictive value of basal P4 on P4 rise on the day of OR, patients with lower P4 on OR day had slightly lower basal P4 values, while patients with higher P4 on OR day (≥ 2 ng/ml) had higher basal P4 levels but not in a statistically significant amount. Venetis et al. showed that ART cycles with higher basal P4 concentrations are more likely to show P4 rise after stimulation^19^. Such a finding could indicate that a jump in P4 on hCG administration days may not be related to stimulation alone; it may be adrenal^29–31^. We also did not find a correlation with GT dose, so we recommend that further studies regarding the progesterone source are needed.

During the menstrual cycle, FSH and LH promote steroid biosynthesis among which is P4 biosynthesis. As FSH and LH act on granulose cells, they, as well as theca cells, produce P4^9^. We found that FSH is a predictor for P4 levels. Huang et al.^32^ found that patients that are younger have lower FSH levels and a longer duration of P4 elevation, so prolonged duration of P4 elevation may occur easily in patients with a better ovarian reserve^32^. In another study that investigated the association of basal levels of E2 and FSH with elevated P4, authors Park et al.^33^ found that this association was not significant.

Different characteristics, such as age and weight, are closely associated with the hormonal status of women and therefore impact fertility^34,35^. However, in our study, none of the tested socio-demographic parameters were confirmed as factors that could be used for prediction of P4 levels on the OR day. These findings are in accordance with other performed multivariate analyses in which, after adjusting for female age, body mass index, smoking status, and previous parity, only sperm quality and P4 levels were significant predictors of ART outcomes^36,37^.

The key finding of our study was that high basal FSH levels can be used as predictors of low P4 levels on the OR day, which is necessary to improve the chances of successful ART. Consequently, our findings are useful when planning ART in clinical practice in general. In the case of either inadequately low basal FSH or high P4 on the OR day, to achieve better fertility rates, the embryo can be frozen and another embryo transfer can be planned for one of the following cycles.

There are some limitations to our study. Firstly, the number of patients in the study could have been larger. Furthermore, the sample sizes of the long and short protocols vary, as there were more patients in the short GnRH antagonist protocol. However, the study included all patients who had the ART procedure at the clinic during a one-year period. Also, as in all centers/clinics, the value and confidence intervals vary with different P4assay kits.

In conclusion, this study found a significant correlation between the levels of P4 on the day of OR and a positive outcome for the ART procedure. Patients with lower levels of P4 (< 2 ng/ml) were more likely to both achieve pregnancy and deliver a vital infant. By examining the association between P4 and basal hormone levels, a significant positive correlation was found between higher AMH values and higher P4 values at OR day, and an inverse correlation was found between lower FSH values and higher P4 values. High basal FSH levels were found to be an important predictor of lower P4 levels (< 2 ng/ml) on OR day.

## Materials and methods

### Patients

A prospective observational study was conducted at the Clinic for Gynecology and Obstetrics Clinical Center of Serbia from January 1st to December 31st, 2015. The study included all patients that were enrolled in the ART procedure at the Clinic during the study period and that fulfilled the inclusion criteria. The inclusion criteria included being 18 to 40 years old and independent (not having a legal guardian); having a BMI of 18 to 30 kg/m^2^, a regular menstrual cycle (25 to 32 days), an established diagnosis of infertility and agreeing to participate in the study. All types of female infertility patients were included in the study. The exclusion criteria included azoospermic male patients or female patients that do not fulfill the inclusion criteria. Infertility was diagnosed and classified according to European Society of Human Reproduction and Embryology (ESHRE) guidelines^38^. For all patients finally included in the study sample socio-demographic data as well as a thorough medical history were taken.

All patients were thoroughly informed about the research and all booked ART procedures and signed the informed consents for inclusion in the study as well as the ART itself. All procedures were carried out in accordance with relevant guidelines and regulations. The recruitment of patients was performed in accordance with the Helsinki declaration. The research was approved by the Ethics Committee of the Faculty of Medicine in Belgrade (25138). ClinicalTrials.gov Identifier: NCT04447677, date of registration 25.06.2020.

### Protocols of stimulation

A detailed explanation of the stimulation protocol and patient monitoring were presented in a previous study^13^. Briefly, patients on a long stimulation protocol with GnRH agonists received Triptoreline (Diphereline, Ipsen Pharma Biotech, France) starting in the mid-luteal phase of the cycle prior to the study. Following pituitary suppression, 2–3 days of the next cycle, ovarian stimulation with gonadotropins (GTs)—recombined FSH (Gonal-F, Serono, Switzerland) was initiated depending on patient age, ovarian reserve, and ovarian response in the previous procedures. Patients on a short stimulation protocol with GnRH antagonists received the same stimulation starting on the 2nd or 3rd day of the cycle. GnRH antagonists (Cetrorelix [Cetrotide, Merk Serono, Germany]) were added when the leading follicle reached a diameter of 14 mm and were administered until the day of hCG trigger. In both COS protocols the ovarian response was followed by sequential transvaginal ultrasound examinations and determination of estradiol levels in the blood. When two or more follicles were > 18 mm in diameter, hCG (Pregnyl, Organon, The Netherlands) was administered.

### ART procedure

The insemination methods were in vitro fertilization (IVF), intracytoplasmic sperm injection (ICSI), or the combined method. Whether fertilization occurred or not was ascertained 16–20 h after insemination by the presence of two pronuclei. In assessing obtained embryo quality, the criteria of the Istanbul Consensus of Clinical Embryologists were used. The criteria include evaluation of the degree of fragmentation and symmetry of the blastomere. Embryos are divided into class A (solid, with 1–10% or without fragmentation, perfect symmetry), class B (medium, 11–25% fragmentation, moderate asymmetry), class C (poor, > 25% fragmentation, pronounced asymmetry)^39^. All evaluations were performed together by our team of three embryologists. Finally, on the second or third day after OR, an embryo transfer was performed under the control of a transabdominal ultrasound. According to current recommendations, we transferred 1–3 embryos in all patients regardless of the P4 group (Table 2). The luteal phase was supported with P4 depo intramuscular administration.

### Hormone measurements

**Figure 1:**
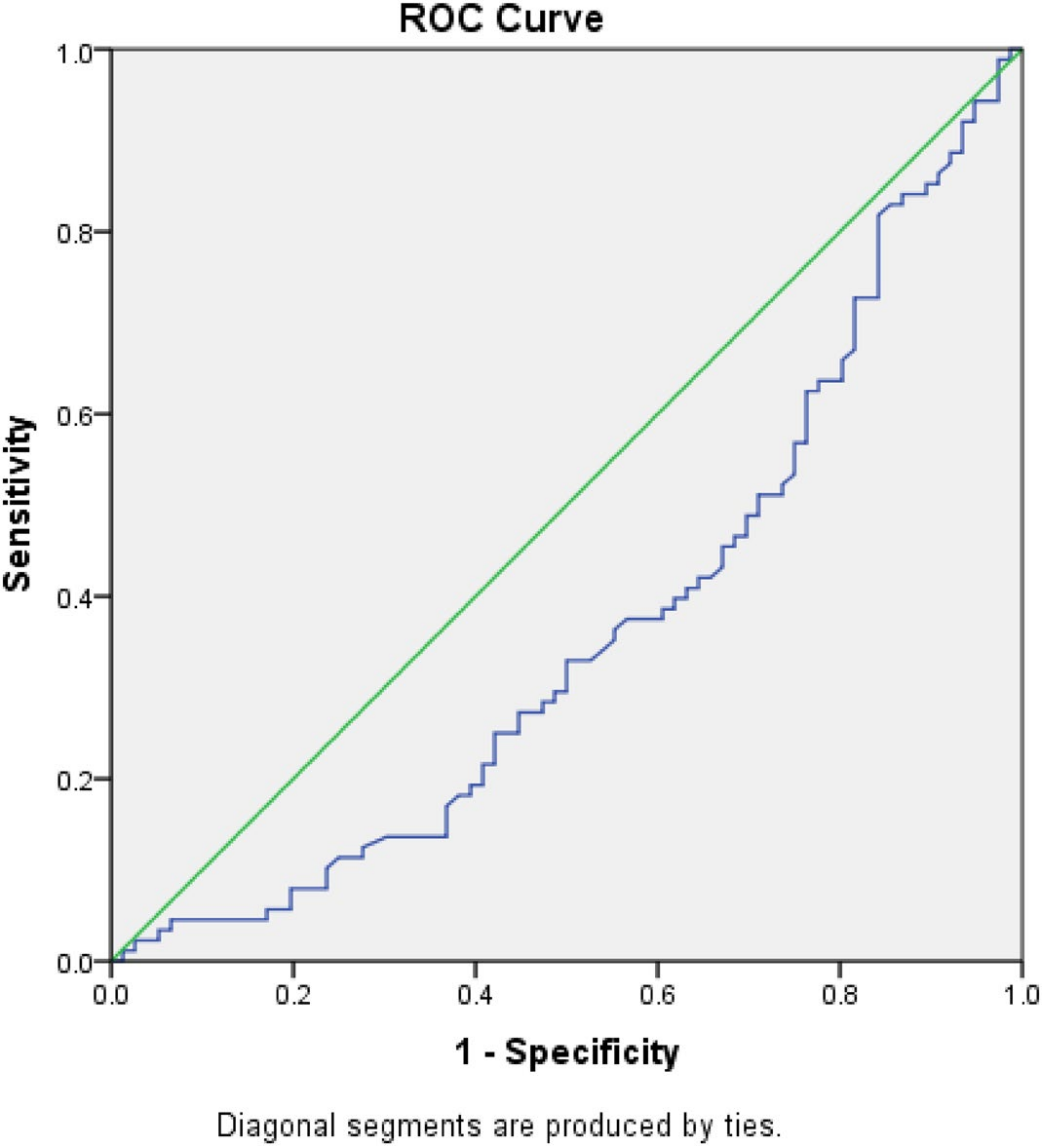
ROC analysis for determination of P4 on OR day effect on achieving pregnancy.

We measured basal serum levels of estradiol (E2), progesterone (P4), follicle-stimulating hormone (FSH), luteinizing hormone (LH), and anti-Mullerian hormone (AMH) for all patients on the 2nd or 3rd day of their cycle, which we always do before stimulation commencement according to classic protocols. The referral range in our laboratory for basal FSH was 3.5 to 12.5 IU/L, for LH 2.4 to 12.6 IU/L, for E2 0 to 160 pg/mL, and for P4 0 to 1.14 ng/mL, while for AMH it was 0.19 to 9.13 ng/ml. Levels of P4 were also measured on the day of OR. Blood samples were taken using Vacutainer tubes (BD Vacutainer Systems) and centrifuged. The measurement methods are explained in our previous study^13^. Briefly, the AMH value in the serum was measured using enzyme-linked immunosorbent assay (ELISA), and other hormones (FSH, LH, E2, and P4) were analyzed using chemiluminescent immunoassay. Intra- and inter-assay precision at concentrations most relevant to the study were 5.9% and 4.7% for FSH, 7.3% and 4.5% for LH, 5.2% and 3.2% for E2, and 4.1% and 2.3% for P4. In our study, we used the Receiver Operating Characteristics (ROC) analysis to estimate the effect of P4 levels on achieving clinical pregnancy. We determined that a cutoff of 2 ng/mL represented the value where the event frequency changes abruptly (sensitivity = 47%; specificity = 69%) (Fig. 1). The obtained cutoff was in accordance with previous literature data (14–16). Therefore, the level of P4 on the OR day was assessed as continuous as well as low (< 2 ng/ml) and high/elevated (≥ 2 ng/ml). Consequently, patients were classified into two groups depending on their P4 levels on the OR day: 1) low P4 (< 2 ng/ml) and 2) P4-high (≥ 2 ng/ml).

### Outcome measures

The main outcome measures included procedure success and pregnancy outcome. Pregnancy was confirmed by a positive finding of serum β-hCG (> 25 MIU/ml) 14 days after embryo transfer. Clinical pregnancies were confirmed by transvaginal ultrasound findings of a gestational sac with a vital embryo at the 6^th^ gestational week. All patients with confirmed pregnancy were regularly followed-up according to current protocols throughout the pregnancy. Based on the gestational week of the pregnancy, end-of-pregnancy outcomes were classified as either miscarriage or delivery (live born infant ≥ 24 weeks of gestation).

### Statistical analysis

Results were presented as a mean ± standard deviation for variables with normal distribution and as a median and interquartile range for variables for which the distribution was not normal. Categorical variables are presented as absolute and relative frequencies. Receiver Operating Characteristics (ROC) analysis was applied to determine the P4 cutoff level that would be used for classification of patient groups (low and high P4). Comparison of the mean values of independent groups of data was performed using ANOVA. For parameters without normal distribution, testing of the significance between groups was performed using the Kruskal–Wallis test. Correlation analysis (Pearson/Spearman) was used to investigate the association of patient and procedure characteristics with P4 levels on OR day. The potential predictive value of tested variables on P4 on OR day was assessed using Univariate and Multivariate logistic regression analyses. A significance of 0.05 was required for a variable to be included in the multivariate model, whereas 0.1 was the cutoff value for exclusion. Odd ratios with the corresponding 95% confidence intervals were estimated. All analysis was performed using the Statistical Package for the Social Sciences 22.0 (SPSS, Inc, Chicago, IL), and differences were considered statistically significant at a probability level of less than 0.05.

## Abbreviations

IVF: In vitro fertilization
ART: Assisted reproductive technologies
OR: Oocyte retrieval
ICSI: Intracytoplasmic sperm injection
BMI: Body mass index
GnRH: Gonadotropin-releasing hormone
GT: Gonadotropin
COS: Controlled ovarian stimulation
LH: Luteinizing hormone
FSH: Follicle stimulating hormone
E2: Estradiol
P4: Progesterone
